# Exploring the Potential for Fungal Antagonism and Cell Wall Attack by *Bacillus subtilis natto*

**DOI:** 10.3389/fmicb.2020.00521

**Published:** 2020-03-31

**Authors:** Anna Schönbichler, Sara M. Díaz-Moreno, Vaibhav Srivastava, Lauren Sara McKee

**Affiliations:** ^1^Division of Glycoscience, Department of Chemistry, KTH Royal Institute of Technology, AlbaNova University Centre, Stockholm, Sweden; ^2^Wallenberg Wood Science Center, Stockholm, Sweden

**Keywords:** biocontrol, *Bacillus subtilis natto*, Chitinase, fungal cell wall, protease, secretome

## Abstract

To develop more ecologically sustainable agricultural practices requires that we reduce our reliance on synthetic chemical pesticides for crop protection. This will likely involve optimized biocontrol approaches – the use of beneficial soil microbes to attack potential plant pathogens to protect plants from diseases. Many bacterial species, including strains of *Bacillus subtilis*, have been explored for their biocontrol properties, as they can control the growth of harmful fungi, often by disrupting the fungal cell wall. A strain that is not often considered for this particular application is *Bacillus subtilis natto*, primarily known for fermenting soybeans via cell wall degradation in the Japanese probiotic dish “natto.” Because deconstruction of the fungal cell wall is considered an important biocontrol trait, we were motivated to explore the possible anti-fungal properties of the *B. subtilis natto* strain. We show that *B. subtilis natto* can use complex fungal material as a carbon source for growth, and can effectively deconstruct fungal cell walls. We found degradation of fungal cell wall proteins, and showed that growth on a mix of peptides was very strong. We also found that intact fungal cell walls can induce the secretion of chitinases and proteases. Surprisingly, we could show that chitin, the bulk component of the fungal cell wall, does not permit successful growth of the *natto* strain or induce the secretion of chitinolytic enzymes, although these were produced during exposure to proteins or to complex fungal material. We have further shown that protease secretion is likely a constitutively enabled mechanism for nutrient scavenging by *B. subtilis natto*, as well as a potent tool for the degradation of fungal cell walls. Overall, our data highlight *B. subtilis natto* as a promising candidate for biocontrol products, with relevant behaviors that can be optimized by altering growth conditions. Whereas it is common for bacterial biocontrol products to be supplied with chitin or chitosan as a priming polysaccharide, our data indicate that this is not a useful approach with this particular bacterium, which should instead be supplied with either glucose or attenuated fungal material.

## Introduction

One of the greatest threats to global food security is the massive loss of staple crops to fungal disease (Dean et al., 2012; Fisher et al., 2012). To try to avoid devastating harvest losses, farmers apply increasing amounts of pesticides (Ng et al., 2019). As a result, synthetic pesticides are often found at high levels in soil and water, and accumulate in food webs with increasing toxicity (Damalas and Eleftherohorinos, 2011; Jorgensen et al., 2012; Silva et al., 2019). This poses risks to environmental and human health, and contributes to the increasing incidence of fungal resistance to chemical pesticides (Deising et al., 2008; Price et al., 2015; Hawkins et al., 2018). It is therefore imperative that we find alternative approaches to crop protection. One popular suggestion is to increase the efficacy of biocontrol, which is the use of living organisms to control plant disease vectors. This includes certain bacteria that are naturally able to limit the growth of phytopathogenic fungi. The design of an effective bacterial biocontrol product is challenging, since a thorough understanding of target, environment, mode of action, and delivery system is needed (O’Brien, 2017). Biocontrol bacteria are often packaged together with chitin, which has long been thought to prime their anti-fungal behaviors (Sid Ahmed et al., 2003; Kokalis-Burelle et al., 2006; Yandigeri et al., 2015).

A bacterial species with well-known biocontrol capability is *Bacillus subtilis*, found in diverse environments, but generally regarded as a soil dweller (Kunst et al., 1997; Earl et al., 2008). When present in the rhizosphere of plants, *B. subtilis* conveys beneficial effects on plant growth, in addition to displaying fungal antagonism, and can limit the growth of phytopathogenic species (Cazorla et al., 2007; Choudhary and Johri, 2009; Kumar et al., 2012; Xiao-ying et al., 2015).

**FIGURE 1:**
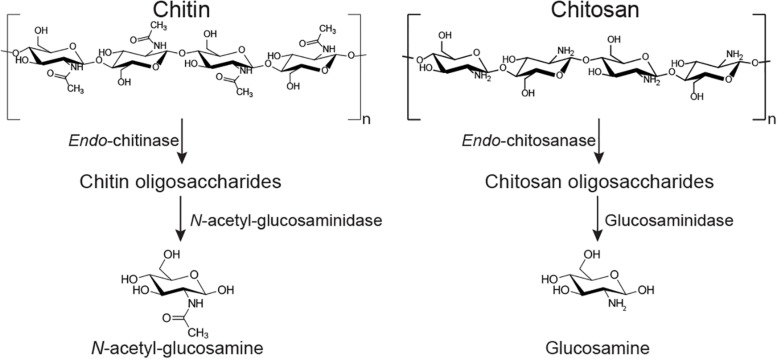
Simplified depiction of two potential chitin degradation pathways. Chitin can be deconstructed to oligosaccharides by an *endo*-chitinase (EC 3.2.1.14); the oligos are converted to monosaccharide GlcNAc by an *exo*-acting *N*-acetylglucosaminidase (EC 3.2.1.30). The (partly) deacteylated form chitosan can be hydrolyzed by an *endo*-chitosanase (EC 3.2.1.132) into oligosaccharides, prior to deconstruction by *exo*-enzymes such as glucosaminidase (EC 3.2.1.165). Most chitin deacetylating enzymes (EC 3.5.1.41) are more active on lower molecular weight chito-oligosaccharides than on very high molecular weight polysaccharides (Zhao et al., 2010).

The ability of *B. subtilis* and other soil bacteria to secrete chitin-degrading enzymes (Figure 1) is often used as a proxy indicator for fungal-antagonistic properties (Sundheim, 1992; Swain et al., 2008; Swain and Ray, 2009; Luo et al., 2015; Veliz et al., 2017). Fungal cell wall deconstruction is viewed as a key element of fungal antagonism and therefore of anti-fungal biocontrol in general (Swiontek Brzezinska et al., 2014; O’Brien, 2017; Veliz et al., 2017). The fungal cell wall consists of a common core of β-1, 3-,β-1,6-D-glucans and chitin. Chitin is a high molecular weight crystalline polysaccharide made of *N*-acetylglucosamine (GlcNAc) residues connected by β-1,4-glycosidic linkages, and it provides strength and toughness to stabilize and protect the cell (Gow et al., 2017). The outer layer of the cell wall typically comprises highly mannosylated glycoproteins (Gow et al., 2017).

The addition of chitin directly into soil can increase soil suppressiveness, likely by causing an increase in the production of bacterial chitinases or other antagonistic metabolites (Cretoiu et al., 2013; Debode et al., 2016). A key question remains to be answered: does the addition of chitin lead to an increase in chitinase activity because of an increase in cell numbers of chitinase-secreting bacteria, or because bacterial cells experience a specific upregulation in expression of chitinase-encoding genes?

*Bacillus subtilis natto* (Qiu et al., 2004; Nishito et al., 2010; Kamada et al., 2014) is a well-known Japanese strain, primarily used in the agricultural setting for food production (Horie et al., 2018; Wang et al., 2018). In the famous natto fermentation process, secreted bacterial proteolytic and glycolytic enzymes transform soybeans into a probiotic food (Kuo et al., 2006; Hu et al., 2010; Lee et al., 2019). Analysis of the published genome of *B. subtilis natto* strain BEST195 (Nishito et al., 2010; Kamada et al., 2014) reveals a number of possible chitin degrading enzymes, including a chitosanase and multiple hexosamininidases (Figure 1). This is in addition to the numerous well-known proteases the species produces (Weng et al., 2017). We were motivated to explore the extent to which *B. subtilis natto* is capable of deconstructing the chitin- and protein-rich fungal cell wall, and whether the bacterium modulates the production of degradative enzymes upon sensing the main components of a fungal cell wall.

We have assayed *B. subtilis natto* for its ability to draw nutrition from complex fungal cell wall material and its three main components – chitin, β-glucan, and protein – using growth analyses, biochemical assay, and proteomic assay of the bacterial secretome. By exploring the factors that can promote classical biocontrol activities in *B. subtilis natto*, we believe that more effective optimized formulations of the bacterium can be developed that have stronger and longer lasting protective effects, thereby improving the cost-efficacy of the technology (Barratt et al., 2018). We found that this strain can indeed metabolize fungal cell walls, and secretes high levels of chitinase and protease activities during growth – but that neither chitin nor β-glucan supported strong growth or elevated enzyme secretion. We propose that pre-cultivating *B. subtilis natto* with fungal cell wall extract – but not isolated chitin polysaccharide – may prime the bacterium for stronger biocontrol behavior in the field.

## Materials and Methods

### Growth Analyses of *B. subtilis natto*

#### Preparation of Growth Substrates

Mushrooms of the species *Agaricus bisporus* were purchased from a grocery store, cut into small pieces, and lyophilized over a weekend. The dried pieces were then ground into a fine powder using a bead mill tissue homogenizer. Fungal cell wall (FCW) was extracted in the form of an alcohol insoluble residue (AIR), according to the method described by Smiderle et al. (2013). Approximately 3 g of fungal powder was incubated overnight in 30 mL of 70% ethanol on a shaking bench. Ethanol was removed by centrifugation for 10 min at 5000 × *g*. The supernatant fluid was discarded, and the remaining pellet was washed in 25 mL 70% ethanol three times. The remaining pellet was then washed by resuspending in 25 mL 100% acetone, and centrifuged twice to remove all supernatant liquid. After the third wash, sample was transferred into an aluminium lined petri dish and left to dry overnight under air flow. The yield of AIR was approximately 30% of the initial *A. bisporus* fruiting body (FB) powder.

#### Strain Maintenance and Growth Rate Analysis

A lyophilized pellet of cultured *B. subtilis natto* (DSM-1092) was purchased from DSMZ (Braunschweig, Germany) and rehydrated in 700 μL LB medium prior to overnight propagation at 30°C in 10 mL LB medium. For long-term storage at −80°C, 500 μL aliquots of liquid culture were added to 200 μL 80% glycerol. To generate detailed growth curves of *B. subtilis natto* provided with different substrates (carbon sources putatively able to induce an anti-fungal effect), 100 μL from 10 mL starting cultures grown overnight in LB medium, were inoculated into 10 mL Spizizen minimal medium (Anagnostopoulos and Spizizen, 1961). 1 L of Spizizen minimal medium contained 2 g (NH_4_)_2_SO_4_, 14 g K_2_HPO_4_, 6 g KH_2_PO_4_, 1 g trisodium citrate dihydrate and 0.2 g MgSO_4_⋅7H_2_O, as well as 0.01 g Yeast Extract and 0.85 mg MnSO_4_ (Hauser and Karamata, 1994). Cultures (10 mL) were additionally supplemented with 50 mg of either *A. bisporus* FB powder, *A. bisporus* FCW, β-chitin (Maharani Chitosan PTV, Ltd., Gujarat, India), the branched fungal β-glucan scleroglucan (Cargill, MN, United States), or peptone (Sigma-Aldrich, MO, United States). For each carbon source tested, three cultures with and three cultures without 0.5% glucose were prepared. Cultures were incubated at 30°C with rotary shaking at 200 rpm. 300 μL samples were taken hourly and absorbance readings (A_600_) were taken after solid material had settled. Control growth experiments provided Spizizen medium with/without glucose and no additional carbon source. Non-inoculated bacterial free cultures (BFC) were also prepared to account for the absorbance deriving from dissolved or dispersed carbon source.

#### Secretome Analysis

##### Collection and washing of secreted proteins

After cultures of *B. subtilis natto* reached stationary phase, cultures were centrifuged (5000 × *g*, 30 min) and cell-free supernatant (CFS) was carefully removed by pipetting into a fresh tube. Control secretome experiments used the CFS from BFC experiments, prepared in the same way. After collecting the CFS, aliquots were washed twice in H_2_O using Amicon Ultra 0.5 Centrifugal Filter Units with 10 kDa nominal molecular weight cut-off (Merck, Darmstadt, Germany). A Bradford Protein Assay was performed to measure the protein concentration. Briefly, Bradford Dye Reagent (Bio-Rad Laboratories, CA, United States) was diluted 1:4 in H_2_O. In a 96-well plate, 10 μL of CFS were mixed with 200 μL of diluted Bradford Dye Reagent and incubated at room temperature for 5 min. Absorbance was measured at 595 nm using a BMG Labtech CLARIOstar spectrophotometer (BMG labtech, CA, United States). Protein concentration was quantified by comparison to a standard curve produced using Bovine Serum Albumin (Bio-Rad Laboratories, CA, United States).

##### Secretome SDS-PAGE

To visualize the proteins present, CFS samples were analyzed via SDS-PAGE. For this, 5 mL of CFS was concentrated to ∼500 μL using Amicon Ultra 0.5 Centrifugal Filter Units with 10 kDa nominal molecular weight cut-off (Merck, Darmstadt, Germany). Approximately equal total amounts of protein were loaded onto an SDS-PAGE gel, dyed using InstantBlue Stain (Sigma-Aldrich, MO, United States).

##### Sample preparation for mass spectrometric analysis

To identify proteins visualized by SDS-PAGE, trypsin in-gel digestion was performed as described previously in Srivastava et al. (2013). Briefly, bands of interest were cut out of the gel and diced into ∼1 mm^3^ pieces. These were incubated in 200 μL gel reagent (50% 0.1 M ammonium carbonate, 50% acetonitrile, pH 10) at 37°C for 60 min. Gel pieces were dehydrated by mixing with 100% acetonitrile. Next, 100 μL of a 10 mM solution of DTT in 50 mM ammonium bicarbonate was added to the gel pieces, and incubated for 30 min at 60°C. After removing and discarding excess supernatant, 30 μL of 1% iodoethanol solution in 50 mM ammonium bicarbonate was added for a 15-minute incubation at 37°C. Dehydration with 100% acetonitrile was repeated. To digest proteins in the gel, 30 μL trypsin solution (comprising trypsin (10 ng/μL) and Protease Max (0.01%) in 50 mM ammonium bicarbonate) was added to dehydrated pieces, for incubation on ice for 15 min. Excess trypsin solution was removed, and 50 μL of 50 mM ammonium bicarbonate solution was added for overnight incubation at 37°C. Supernatant containing released peptides was transferred to a fresh tube. Additional peptides were extracted from the gel pieces using 50 μL formic acid solution (5% formic acid, 50% acetonitrile, 45% H_2_O), and incubated for 5 min at room temperature. Peptides were speed-vacced to dryness and stored at −20°C until analysis by mass spectrometry.

##### Mass spectrometric analysis

Peptide analysis and identification was performed as described by Srivastava et al. (2013). A reversed-phase liquid chromatography electrospray ionization mass spectrometer (LC-ESI-MS/MS), using a nanoACQUITY ultra-performance liquid chromatography (UPLC) system coupled to a Q-TOF mass spectrometer (Xevo Q-TOF; Waters, Milford, MA, United States) was used. Briefly, peptides were loaded onto a C18 trap column (Symmetry 180 μm × 20 mm, 5 μm; Waters, Milford, MA, United States) followed by washing with 1% (v/v) acetonitrile and 0.1% (v/v) formic acid at 10 μL min^–1^ for 5 min. The samples eluted from the trap column were separated on a C18 analytical column (75 μm × 100 mm, 1.7 μm; Waters, Milford, MA, United States) at 250 nl min^–1^ using 0.1% formic acid as solvent A and 0.1% formic acid in acetonitrile as solvent B in a stepwise gradient: 0.1%–10% B (0–5 min), 10–30% B (5–32 min), 30–40% B (32–35 min), 40–85% B (36–38 min), 85% B (38–40 min), 85–0.1% B (40–42 min), and 0.1% B (42–60 min). The eluting peptides were sprayed in the mass spectrometer (capillary and cone voltages set to 2.1 kV and 35 V, respectively), and MS/MS spectra were acquired using automated data-directed switching between the MS and MS/MS modes using the instrument software (MassLynx V4.0 SP4). The three most abundant signals of a survey scan (400–1600 m/z range, 1 s scan time) were selected by charge state, and collision energy was applied accordingly for sequential MS/MS fragmentation scanning (50–1800 m/z range, 1 s scan time). The MS raw data files were processed using Mascot Distiller (version 2.4.3.2, Matrix Science, London, United Kingdom) and the resulting files were submitted to a local Mascot (Matrix Science, version 2.3.1) server using the NCBI database with both *B. subtilis* (1084562 sequences) and general fungi (5915770 sequences) taxonomies. The following settings were used for the database search: trypsin-specific digestion with one missed cleavage allowed, ethanolylated cysteine as fixed and oxidized methionine as variable modifications, peptide tolerance of 100 ppm, and fragment tolerance of 0.6 Da. Peptides with Mascot ion scores exceeding the threshold for statistical significance (*p* < 0.05) were approved. Only proteins identified by two or more unique peptides were selected.

#### Biochemical Assays

##### Chitosanase assay

The blue colored substrate azurine cross-linked (AZCL)-chitosan (Megazyme, Co., Wicklow, Ireland; Cat. No. I-AZCHANF) was used according to the manufacturer’s instructions to screen secretomes for *endo*-chitosanase activity (Huang et al., 2017). 1 mL of washed CFS was mixed with 1 mL AZCL-chitosan (2 g L^–1^ in H_2_O) and incubated at 30°C with rotary shaking. The negative control contained 1 mL AZCL-chitosan and 1 mL washed non-inoculated culture medium. Absorbance at 590 nm was measured after 15–20 h of incubation.

##### Protease assay

To quantify protease activity in the supernatant of *B. subtilis natto*, a Pierce Protease Assay Kit (Thermo Scientific, Rockford, United States) was used according to the manufacturers’ instructions (Rao et al., 1997; Tian et al., 2004). Briefly, 100 μL of succinylated casein solution was added into microplate wells. 50 μL of CFS was added on this, and the microplate was incubated for 20 min at 27°C. After the incubation, 50 μL of 2,4,6-trinitrobenzene sulfonic acid (TNBSA) working solution was added to the wells, and the plate was allowed to develop for 20 min in the dark at room temperature. Absorbance was then measured at 450 nm in a plate reader (CLARIOstar, BMG Labtech). Control experiments (CFS without succinylated casein) were subtracted from the measured absorption. Trypsin activity (Thermo Scientific) against the same substrate was used as a general protease standard.

##### Assays using labeled monosaccharide substrates

To measure GlcNAcase activity in the secretome of *B. subtilis natto*, pNP assays with 4-Nitrophenyl β-D-*N*-acetylglucosamine (Both Sigma-Aldrich, MO, United States) substrates were performed. For this, the substrate was dissolved in filtered H_2_O to achieve a 10 mM stock concentration. To achieve complete dissolution, up to 3% DMSO was added. To prepare the assay, 20 μL sodium phosphate buffer (pH 6.5) was added to wells of a 96 well plate. To this was added 20 μL of substrate (resulting in a final concentration of 1 mM) and 50 μL of CFS, brought to a final volume 200 μL with H_2_O. For each type of CFS tested, a substrate blank, containing no pNP substrate, was also performed. The plate was incubated at 37°C for 6 h with absorbance measured at 410 nm in a plate reader (CLARIOstar, BMG Labtech) every hour. The amount of pNP produced was quantified by reference to a pNP standard curve.

To measure GlcNase activity in the secretome of *B. subtilis natto*, an assay with 4-Methylumbelliferyl (MU) β-D-Glucosaminide (Carbosynth, Compton, United Kingdom) substrate was performed. For this, the substrate was dissolved in water to a stock concentration of 6 mM. The assay was prepared as described above, using a final substrate concentration of 0.6 mM. The plate was incubated at 37°C for 6 h, and the release of fluorescent 4MU was measured in a BMG Labtech CLARIOstar fluorometer (BMG labtech, CA, United States) using a 340–380 nm bandpass excitation filter and a 455–465 nm bandpass emission filter. The amount of 4MU produced was quantified by reference to a 4MU standard curve.

## Results and Discussion

### Fungal Cell Walls – But Not Chitin – Are a Strong Growth Substrate for *B. subtilis natto*

We first investigated the ability of *B. subtilis natto* to grow on fungal fruiting body (FB), extracted fungal cell wall (FCW), and the two major FCW carbohydrates – chitin and β-glucan – as sole carbon source. We also studied growth on a standard mix of soluble proteins (peptone), to serve as a mimic for the ill-defined protein component of FCW and other chitinous materials. The bacterium was incubated in minimal medium containing 5 g L^–1^ carbon source. Control experiments provided only minimal medium with no carbon source. All conditions were analyzed in triplicate. Cultures were sampled regularly for up to 26 h, and optical density (OD) was measured as an indicator of culture turbidity. In addition, all of these conditions were studied both with and without the additional presence of 5 g L^–1^ glucose. This experimental set-up allowed us to investigate the potential for degradation of FCW and its components even when not being used as the primary carbon source supporting growth (i.e., the potential for co-metabolism of FCW components).

**FIGURE 2:**
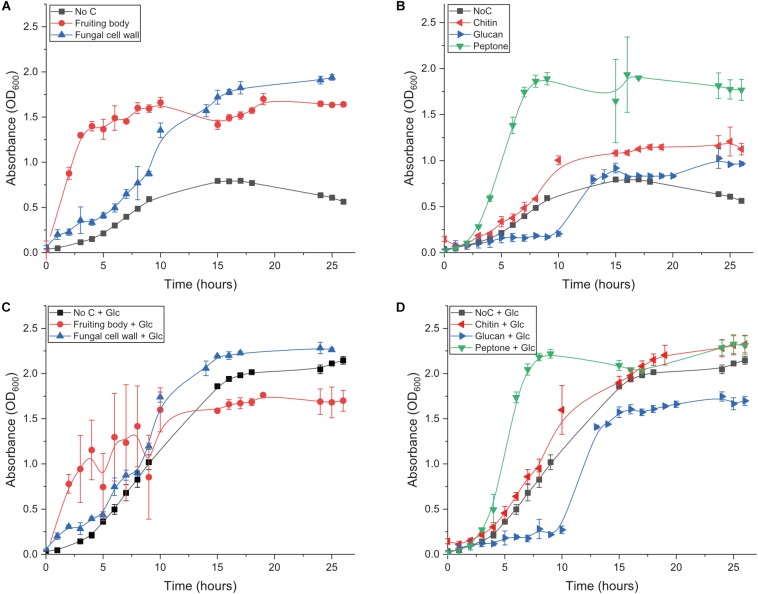
Growth behavior of *B. subtilis natto* growing on different substrates. **(A)** Growth of *B. subtilis natto* on complex carbon sources: fungal fruiting body and fungal cell wall. **(B)** Growth of *B. subtilis natto* on isolated components of the fungal cell wall: β-chitin, β-glucan (scleroglucan), and peptone. **(C)** Growth of *B. subtilis natto* on fungal fruiting body and fungal cell wall, in medium additionally supplemented with glucose. **(D)** Growth of *B. subtilis natto* on β-chitin, β-glucan (scleroglucan), and peptone, in medium additionally supplemented with glucose. In all experiments, the bacterium was inoculated from LB starter cultures into 10 mL Spizizen minimal medium, additionally containing 50 mg of the carbon source being tested. OD_600_ was measured regularly for up to 26 h. Growth curves performed without experimental carbon source are included in both panels, labeled as No C. Raw absorbance data are provided for all growth curves in Supplementary Tables S1–S6.

Growth in medium without glucose or any other experimental carbon source was very slow and reached a low final OD (Figure 2 and Table 1): the small amount of yeast extract in the un-supplemented minimal medium accounts for the low amount of growth that was achieved. Growth on glucose as sole carbon source was strong (Table 1 and Figure 2C). Only two experimental carbon sources permitted an increase in the baseline growth level of glucose cultures. Cultures supplemented with glucose and FCW reached a higher final OD than could be achieved on just glucose, while cultures supplemented with glucose and peptone showed a much more rapid doubling rate than during growth on only glucose (Table 1 and Figure 2C). This might indicate that only FCW and peptone are being used as carbon source instead of or as well as the glucose that is also present, while in all other glucose-supplemented cultures, it was only the glucose supporting bacterial growth, not the experimental carbon source. Growth experiments were repeated in the absence of glucose to test this theory.

**TABLE 1:** Summary of the growth behavior of *B. subtilis natto*.

	**Relative doubling rate**	**Maximum OD achieved**
No carbon source + Glucose	1	2.15
Fungal fruiting body + Glucose	0.68	1.76
Fungal cell wall + Glucose	1.29	2.29
β-chitin + Glucose	0.83	2.33
β-glucan + Glucose	2.52	1.75
Peptone + Glucose	2.81	2.33
No carbon source	0.63	0.80
Fungal fruiting body	2.89	1.70
Fungal cell wall	0.91	1.94
β-chitin	0.80	1.21
β-glucan	1.32	1.02
Peptone	2.43	1.81

*The bacterium was cultivated in Spizizen minimal medium with or without glucose, containing carbon source at 5 g L^–1^. OD_600_ was measured hourly over the course of 26 h. Doubling rates were normalized by comparing to bacterium growing on glucose and no other carbon source.*

Cultures lacking glucose showed that *B. subtilis natto* can indeed use fungal FB and especially FCW as a sole carbon source, showing a rapid doubling rate and reaching a high final OD (Figure 2A and Table 1). However, growth was very poor when the strain was provided with the main glycan components of the FCW, β-glucan and chitin (Figure 2B). Indeed, the fungal β-glucan scleroglucan supported the least growth of all carbon sources tested, and the concentration of secreted proteins was barely measurable; as a result, no further experiments were performed using that carbon source. Peptone as sole carbon source supported rapid growth (Figure 2B), perhaps indicating that proteins are a main metabolic focus for *B. subtilis natto*, explaining why the major glycan components of FCW fail to support strong growth (Table 1). Indeed, the ability of this strain to degrade proteins is quite well known and is exploited in the natto food production process, but we here show that this activity permits the strain to use peptides a sole carbon source during growth.

### Fungal Cell Walls - but Not Chitin – Induce the Secretion of Chitin-Degrading Enzymes

Although growth on chitin was poor, a survey of the *B. subtilis natto* BEST195 strain reference genome had given several indications that chitin degradation by the strain is possible, as we found that it encodes a chitosanase, multiple de-acetylases, and several hexosaminidases/glucosaminidases (Qiu et al., 2003; Nishito et al., 2010; Kamada et al., 2014). In addition, chitin degradation is a focus of much biocontrol literature, and is often taken as a proxy for fungal antagonism (Veliz et al., 2017). During cultivation of *B. subtilis natto* on chitin, we observed a visible increase in dispersibility of the substrate, in terms of smaller particle size and a reduced tendency to settle at the bottom of the flask. This would imply that chitin is in fact being deconstructed, but the poor growth indicates that the bacterium is not readily able to take up the released chito-oligosaccharides, and/or is unable to metabolize those released sugars for energy. Analogy could be drawn with soil bacteria that are effective at deconstructing some types of β-glucan, but which show poor growth on these substrates due to an inability to take up the products of glucan deconstruction (McKee et al., 2019).

**FIGURE 3:**
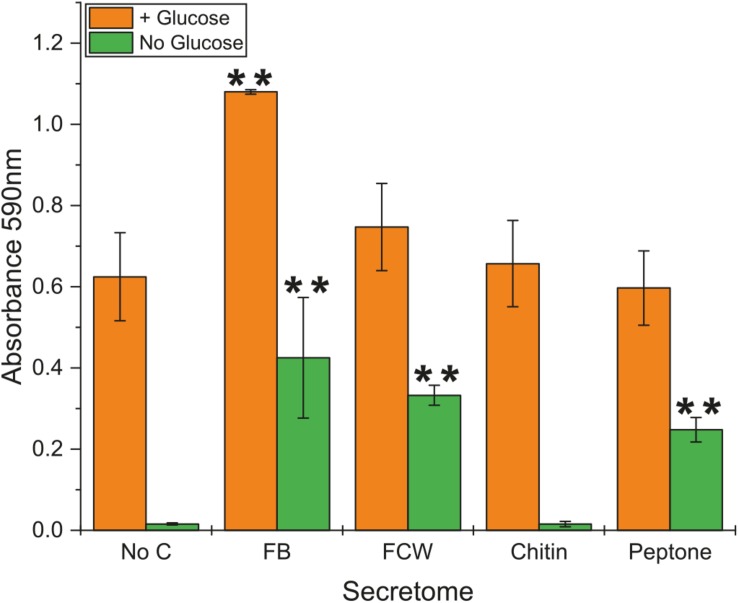
Chitosanase activity in the secretome of *B. subtilis natto*. *B. subtilis natto* was grown on various carbon sources (FB, fungal fruiting body; FCW, fungal cell wall extract), and the culture supernatant containing secreted proteins was assayed for chitosanase activity. Absorbance values (OD_590_) shown were obtained from secretomes incubated with AZCL-chitosan for 15 h. A Student’s *T*-test was performed on the “+Glucose” and separately on the “No Glucose” experiments, showing significant differences from the No C control at the *p* ≤ 0.05 level (indicated by **).

Due to the high background content of sugar in protein secretomes, even after repeated washing with water, we were unable to satisfactorily assay for chitin degradation. However, using a simple colorimetric assay, we were able to investigate the level of chitosanase activity in culture supernatants (secretomes) of *B. subtilis natto* (Figure 3) – chitosan is a de-acetylated form of chitin that can serve as an effective substrate for *endo*-acting chitosanase enzymes (Figure 1). No activity was detected from cultures grown on minimal medium lacking glucose or any carbon source. Perhaps surprisingly, the addition of chitin into this glucose-free growth medium did not lead to any chitosanase activity, suggesting that this carbohydrate does not induce a degradative pathway. The same was true for glucose-free cultures grown with β-glucan. However, the inclusion of glucose in the medium did lead to detectable levels of secreted chitosanase activity, perhaps simply be causing an elevated protein concentration in the culture medium. Among the glucose-containing cultures, only fungal FB led to an increase in chitosanase activity (Figure 3). There was no such increase in cultures grown on FCW, chitin, or peptone. It is more interesting and useful to compare activity profiles of the cultures grown without glucose, where cells are forced to use the experimental carbon source for nutrition. Here we see that chitosanase activity is found in glucose-free cultures containing FB, FCW, or peptone. There is no activity in cultures grown on chitin, or grown without an experimental carbon source. This strongly indicates that complex fungal material and proteins – but not chitin itself – can induce the secretion of chitosanase activity. This assay measures absorbance values after enzyme incubation, but is not a directly quantitative measure of the release of a specific reaction product. As such, it was not feasible to accurately normalize these absorbance data by secretome protein concentration, so it is still possible that the increased chitosanase activity in fungal FB, FCW, and peptone cultures can simply be explained by a higher overall protein content in those secretomes, rather than the specific upregulation of chitosanase gene expression.

**FIGURE 4:**
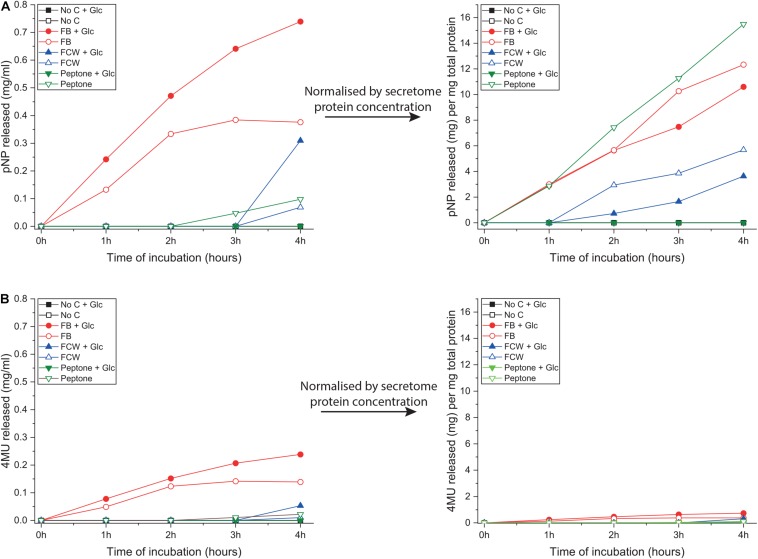
Glucosamine and *N*-acetylglucosamine degradation activity in the supernatant of *B. subtilis natto. B. subtilis natto* was grown on various carbon sources (FB, fungal fruiting body; FCW, fungal cell wall extract), and the culture supernatant containing secreted proteins was assayed for activity using two model substrates: 4NP-GlcNAc and 4MU-GlcN. Cultures grown on chitin or glucan showed no detectable activity on either substrate, so those data are omitted from this figure. **(A)** Hydrolysis of 4NP-GlcNAc by *B. subtilis natto* secretome, after 4 h of incubation. **(B)** Hydrolysis of 4MU-GlcN by *B. subtilis natto* secretome, after 4 h of incubation.

As discussed in the introduction, chitin can be degraded via two complementary pathways (Figure 1). To explore other potential routes to chitin deconstruction, we used two model substrates to screen for activity in the *B. subtilis natto* secretome: 4-nitrophenyl-β-D-*N-*acetylglucosamine (4NP-GlcNAc) and 4-methylumbelliferyl-β-D-glucosamine (4MU-GlcN). These assays respectively, screen for the ability to deconstruct oligosaccharides of chitin (GlcNAc oligos) and chitosan (GlcN oligos), and verified that *B. subtilis natto* can indeed deconstruct both GlcN and GlcNAc oligosaccharides. Washed culture secretome was incubated with one of the two substrates for 4 h, and activity was monitored by absorbance or fluorescence measurements every hour, producing a time-curve of activity (Figure 4). Using standard curves of 4NP and 4MU, we could convert these measurements to concentrations of reaction product released, and this could be normalized by secretome protein concentration.

Hydrolysis of 4NP-GlcNAc and 4MU-GlcN was only detected in cultures grown on peptone or one of the fungal substrates FB and FCW, indicating that enzyme production is being specifically activated by these substrates (Figure 4). As shown in Figure 4A, the highest levels of activity against 4NP-GlcNAc were found in FB secretomes, regardless of the presence or absence of glucose in culture medium. However, after normalizing for total secretome protein concentration, the highest levels of 4NP-GlcNAc activity per mg of secreted protein were found in peptone-induced secretomes. This suggests that, although there is more of the GlcNAc-ase enzyme(s) in the FB cultures, which had a higher total protein content than most other secretomes, there was a higher proportion of GlcNAc-ase enzyme(s) in the peptone-induced secretomes. This implies that there is a specific upregulation of gene(s) encoding GlcNAc-ase enzyme(s) during growth on peptone. To verify these indications, we attempted to quantify gene expression using qPCR. Despite repeated attempts, we were ultimately not able to find any reference genes that were stably expressed in all conditions tested.

Figure 4B shows that hydrolysis of 4MU-GlcN was generally to a far lower level than for the GlcNAc substrate, but again the highest levels of activity were in the FB secretomes. Normalizing these activity data by total secretome protein concentration shows no obvious differences between the different carbon sources, suggesting that there is no activation of specific regulatory mechanisms, such as enhanced gene expression, that would lead to a higher level of GlcN-ase enzyme secretion.

### Proteases Are Secreted in All Conditions, and Enable Degradation of Fungal Cell Wall Proteins

**FIGURE 5:**
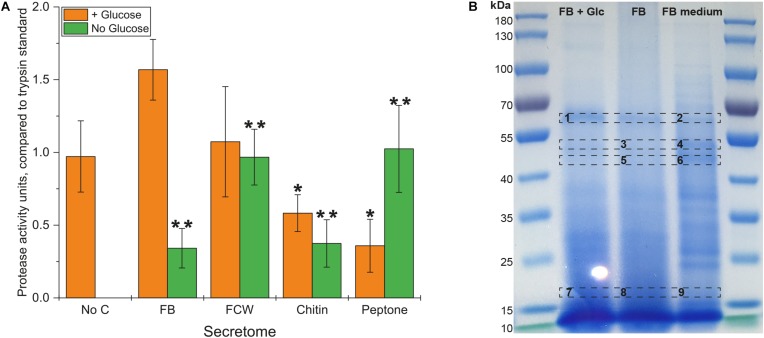
Protease activity in the secretome of *B. subtilis natto*. **(A)** *B. subtilis natto* was grown on various carbon sources (FB, fungal fruiting body; FCW, fungal cell wall extract), and the culture supernatant containing secreted proteins was assayed for protease activity, comparing to the activity of a trypsin standard. A Student’s *T*-test was performed on the “+ Glucose” and separately on the “No Glucose” experiments, showing significant differences from the No C control at the *p* ≤ 0.05 level (indicated by **) and the *p* ≤ 0.1 level (indicated by *). **(B)** FB secretomes were concentrated and analyzed by SDS-PAGE. Proteins in the *B. subtilis natto* FB secretome (gel lanes FB + Glc and FB) were compared with the profile of proteins found in FB medium not inoculated with bacterium, which contained only proteins deriving from the fungal material itself.

Inspired by the strong growth of *B. subtilis natto* on peptone – and previous research indicating that protein digestion is integral to the natto food production process – we assayed our bacterial secretomes for protein degradation. An assay kit was used that compares proteolytic activity to that of purified trypsin enzyme, and showed that protease activity occurred in all glucose-supplemented cultures (Figure 5A). As with the earlier chitosanase activity test, only the FB-supplemented secretomes showed an increased level of protease activity. Comparing results from the no-glucose secretomes shows that the highest levels of protease activity are produced during growth on peptone and FCW, with somewhat lower levels of activity produced during growth on FB and even chitin. The secretion of proteases when proteins are present to be metabolized is logical, but the presence of these activities even during growth on glucose or chitin as sole carbon source suggests that more complex mechanisms are at play. It may be that several proteases are expressed in response to different carbon sources, in order to scavenge for proteins to metabolize. This would fortuitously permit the degradation of FCW when present, which is highly relevant to biocontrol and may largely explain the strong bacterial growth on fungal FB and FCW. According to the MEROPS Peptidase database^1^ (Rawlings et al., 2017), the published genome of *B. subtilis natto* strain BEST195 (Nishito et al., 2010; Kamada et al., 2014) encodes 615 known or putative peptidases. Our own analysis of the predicted proteome of *B. subtilis natto* reveals nine enzymes predicted to be peptidases or proteases that are also predicted to be secreted into the extracellular space. Expression of one or more of these enzymes may be triggered during growth on glucose or chitin, but the precise mechanisms involved are still unknown.

Analysis of culture supernatants by SDS-PAGE (Figure 5B) showed that Spizizen medium supplemented with FB and not inoculated with any bacterium already contained a large number of proteins, deriving from the fungal material itself. Using proteomic mass spectrometry (MS), we could directly compare the profile of proteins in FB-supplemented medium with and without bacterial inoculation (Figure 5B). Several protein bands in the non-inoculated FB medium were reduced or absent following bacterial cultivation, while other bands became (more) visible after cultivation. Sections of the gel with the most visually apparent differences (indicated on Figure 5B) were excised from the gel, subjected to trypsin hydrolysis, and analyzed by MS. Table 2 summarizes the proteins found following bacterial cultivation on FB. A full list of proteins identified in our MS experiments is provided in Supplementary Tables S7, S8. Our analyses verified that a large number of proteins from the fungal material were degraded following incubation with growing cells of *B. subtilis natto*, as far fewer fungal proteins could be detected in equivalent samples after bacterial inoculation (Table 2 and Supplementary Tables S7, S8). Importantly, many of the *B. subtilis natto* proteins identified from our analyses as having been produced during growth on FB are predicted to be proteases. This supports our biochemical and growth data indicating that protease secretion is occurring in these growth conditions, and that these secreted proteases contribute to FCW deconstruction by hydrolyzing the protein component of FCW. This protein hydrolysis was likely a major contributor to the strong bacterial growth observed in FB and FCW cultures, as well as contributing to the visible increase in dispersibility of FCW particles during cultivation, as the strong network of the FCW was disrupted by protein degradation.

**TABLE 2:** Proteins secreted by *B. subtilis natto* during growth on fungal fruiting body.

***B. subtilis natto* culture secretome**	**Non-inoculated medium**
**Band 1: 55–70 kDa**	**Band 2: 55–70 kDa**
Peptide-binding protein Dihydrolipoyl dehydrogenase Peptidase G2 Cytosol aminopeptidase	No bacterial proteins detected
**Band 3: ∼50–55 kDa**	**Band 4: ∼50–55 kDa**
*2 fungal proteins detected* Peptidase M42 Cell wall-associated protease precursor/Peptidase S8 Gamma-glutamyltransferase Bacillopeptidase F Dihydrolipoyl dehydrogenase	*21 fungal proteins detected* No bacterial proteins detected
**Band 5: ∼45 – 55 kDa**	**Band 6: ∼45 – 55 kDa**
*4 fungal proteins detected* Peptidase M28 Cell wall-associated protease precursor Aminopeptidase Extracellular protease vpr, partial Dihydrolipoyl dehydrogenase Gamma-glutamyltransferase Major capsid protein Hypothetical protein	*16 fungal proteins detected* No bacterial proteins detected
**Bands 7 and 8: ∼16 – 20 kDa**	**Band 9: ∼16 – 20 kDa**
DNA starvation/stationary phase protection protein Superoxide dismutase Cell wall-associated protease precursor/Peptidase S8 Serine hydroxymethyltransferase Gamma-glutamyltranspeptidase, Glutathione Hydrolase DNA starvation/stationary phase protection protein Serine hydroxymethyltransferase Aconitate hydratase 1 Glucose-6-phosphate isomerase Triscatecholate Siderophore Binding Protein Acireductone dioxygenase Phage-like element PBSX protein XkdM Oligoendopeptidase F Pectate Lyase	No bacterial proteins detected

*See Supplementary Tables S7, S8 for a detailed description of all proteins identified in all proteomic experiments.*

Due to the literature indicating that chitin can promote suppression of fungal pathogens by soil bacteria (Cretoiu et al., 2013; Debode et al., 2016), many biocontrol products are packaged with chitin as a priming agent. In the case of *B. subtilis natto*, however, our data indicate that such an approach would not encourage strong growth or an increase in FCW degradation. If *B. subtilis natto* is to be exploited as a biocontrol product, an alternative approach could be to use killed (attenuated) fungus as a priming agent in biocontrol products using *B. subtilis natto* to promote the secretion of chitin-, protein-, and FCW-degrading enzymes.

## Conclusion and Outlook

We have demonstrated that the industrial strain *B. subtilis natto* is able to use complex fungal fruiting body and fungal cell wall as a carbon source, and that during growth on this material the species secretes chitin- and protein-degrading enzyme activities. We found conclusively that chitin – the bulk polysaccharide component of fungal cell walls, and a compound often used to stimulate biocontrol activities in soil bacteria – does not support growth of *B. subtilis natto*, nor does it induce the secretion of chitin-degrading activities. Through biochemistry and protein mass spectrometry, we have shown that a strong level of protease activity is produced in most conditions, confirming that this is the primary means by which *B. subtilis natto* draws nutrition from fungal cell walls. Further study is needed to verify that protein degradation in the cell wall can inhibit fungal growth.

## Data Availability Statement

All datasets generated for this study are included in the article/Supplementary Material.

## Author Contributions

AS and LM designed the study. AS performed the experimental work, with contributions from SD-M and VS. LM supervised the project. AS and LM wrote the manuscript, with input from SD-M and VS.

## Conflict of Interest

The authors declare that the research was conducted in the absence of any commercial or financial relationships that could be construed as a potential conflict of interest.

## Footnotes

1 https://www.ebi.ac.uk/merops/
