# Supplementary material for: Exploring the Potential for Fungal Antagonism and Cell Wall Attack by Bacillus subtilis natto
Source: Front Microbiol. 2020 Mar 31;11:521. doi: 10.3389/fmicb.2020.00521 (PMC7136451; doi:10.3389/fmicb.2020.00521)
Supplement: Supplementary file 1 [file Data_Sheet_1.docx]

Supplementary Material

# Supplementary Data

**Table S1: OD_600_ values of *B. subtilis natto* growing in glucose rich and glucose poor medium over 26 hours.**

|  | Growth data for cultures without experimental carbon source | | | | | |
| --- | --- | --- | --- | --- | --- | --- |
| Hours | Glucose + | | | Glucose - | | |
| 0 | 0.0383 | 0.0281 | 0.0409 | 0.0418 | 0.0392 | 0.0386 |
| 1 | 0.0353 | 0.0503 | 0.0505 | 0.0541 | 0.0465 | 0.0462 |
| 3 | 0.1557 | 0.1195 | 0.1549 | 0.1101 | 0.1114 | 0.1217 |
| 4 | 0.2296 | 0.1736 | 0.2309 | 0.157 | 0.1502 | 0.1474 |
| 5 | 0.3605 | 0.3598 | 0.3612 | 0.2115 | 0.2227 | 0.2077 |
| 6 | 0.5163 | 0.4348 | 0.5383 | 0.2972 | 0.2948 | 0.3096 |
| 7 | 0.7303 | 0.5774 | 0.7344 | 0.4194 | 0.3884 | 0.3829 |
| 8 | 0.8882 | 0.7287 | 0.861 | 0.4706 | 0.481 | 0.5069 |
| 9 | 1.0566 | 1.0744 | 0.9249 | 0.5886 | 0.6064 | 0.5858 |
| 15 | 1.8579 | 1.8625 | 1.8601 | 0.7893 | 0.8017 | 0.7916 |
| 16 | 1.9344 | 1.939 | 1.9486 | 0.7763 | 0.7993 | 0.7915 |
| 17 | 1.9733 | 1.978 | 1.9908 | 0.7942 | 0.7929 | 0.8002 |
| 18 | 1.999 | 2.0057 | 2.0424 | 0.7945 | 0.7571 | 0.7572 |
| 24 | 1.9942 | 2.0887 | 2.057 | 0.6453 | 0.6396 | 0.6211 |
| 25 | 2.0912 | 2.1055 | 2.1369 | 0.6091 | 0.633 | 0.5829 |
| 26 | 2.1081 | 2.1902 | 2.1402 | 0.563 | 0.5719 | 0.5532 |

**Table S2: OD_600_ values of *B. subtilis natto* growing in glucose rich and glucose poor medium additionally supplemented with fungal fruiting body over 26 hours.**

|  | Growth data for cultures grown with fungal fruiting body | | | | | |
| --- | --- | --- | --- | --- | --- | --- |
| Hours | Glucose + | | | Glucose - | | |
| 0 | -0.0544 | 0.0166 | -0.038 | -0.0968 | 0.0947 | 0.0751 |
| 2 | 0.8344 | 0.8449 | 0.6616 | 0.944 | 0.881 | 0.8077 |
| 3 | 1.1834 | 1.1318 | 0.5186 | 1.2985 | 1.327 | 1.2726 |
| 4 | 1.3444 | 1.3459 | 0.7734 | 1.3429 | 1.4063 | 1.447 |
| 5 | 1.4628 | 1.6099 | -0.837 | 1.3011 | 1.3002 | 1.4917 |
| 6 | 1.5692 | 1.5824 | 0.7353 | 1.4053 | 1.418 | 1.6461 |
| 7 | 1.6263 | 1.586 | 0.4952 | 1.4388 | 1.4433 | 1.4748 |
| 8 | 1.6784 | 1.6706 | 0.8994 | 1.6267 | 1.6426 | 1.5297 |
| 9 | 1.6813 | 1.7169 | -0.837 | 1.5849 | 1.5571 | 1.6398 |
| 10 | 1.7273 | 1.7481 | 1.318 | 1.6335 | 1.6225 | 1.7275 |
| 15 | 1.6097 | 1.5869 | 1.5679 | 1.453 | 1.4345 | 1.3571 |
| 16 | 1.715 | 1.6537 | 1.6076 | 1.4671 | 1.4764 | 1.5316 |
| 17 | 1.675 | 1.7293 | 1.6085 | 1.4967 | 1.4904 | 1.5641 |
| 18 | 1.6953 | 1.6415 | 1.7145 | 1.5423 | 1.6045 | 1.568 |
| 19 | 1.7606 | 1.7472 | 1.779 | 1.6316 | 1.751 | 1.7139 |
| 24 | 1.7779 | 1.7636 | 1.5265 | 1.6346 | 1.6275 | 1.6786 |
| 25 | 1.8013 | 1.753 | 1.4868 | 1.6157 | 1.6352 | 1.6522 |
| 26 | 1.7686 | 1.7636 | 1.5647 | 1.6185 | 1.6323 | 1.6694 |

**Table S3: OD_600_ values of *B. subtilis natto* growing in glucose rich and glucose poor medium additionally supplemented with fungal cell wall extract over 26 hours.**

|  | Growth data for cultures grown with fungal cell wall extract | | | | | |
| --- | --- | --- | --- | --- | --- | --- |
| Hours | Glucose + | | | Glucose - | | |
| 0 | 0.05 | 0.0581 |  | 0.0622 | 0.0781 | 0.0449 |
| 1 | 0.2086 | 0.231 | 0.1613 | 0.1594 | 0.2612 | 0.1801 |
| 2 | 0.3191 | 0.295 |  | 0.2623 | 0.1935 | 0.2234 |
| 3 | 0.304 | 0.2093 | 0.3372 | 0.3178 | 0.5224 | 0.2357 |
| 4 | 0.4142 | 0.3931 | 0.3752 | 0.3562 | 0.2986 | 0.3482 |
| 5 | 0.4471 | 0.3921 | 0.4637 | 0.4309 | 0.3885 |  |
| 6 | 0.845 | 0.746 | 0.649 | 0.5094 | 0.4441 | 0.5311 |
| 7 | 0.9405 | 0.8488 | 0.8396 | 0.6077 | 0.6381 | 0.701 |
| 8 | 0.8825 | 0.9199 |  | 0.6633 | 0.6629 | 0.983 |
| 9 | 1.2238 | 1.2079 | 1.1542 | 0.8439 | 0.889 | 0.8832 |
| 10 | 1.6831 | 1.7404 | 1.7948 | 1.2967 | 1.4112 |  |
| 14 | 2.115 | 2.0002 |  | 1.5268 | 1.5364 | 1.6482 |
| 15 | 2.1854 | 2.2007 |  | 1.6393 | 1.7294 | 1.7933 |
| 16 | 2.1692 | 2.2287 |  | 1.8069 | 1.7713 | 1.7506 |
| 17 | 2.2385 | 2.2158 |  | 1.855 | 1.8718 | 1.7461 |
| 24 | 2.2828 | 2.3463 | 2.2189 | 1.8612 | 1.922 | 1.9445 |
| 25 | 2.2723 | 2.2769 | 2.239 | 1.9148 | 1.9273 | 1.9732 |

**Table S4: OD_600_ values of *B. subtilis natto* growing in glucose rich and glucose poor medium additionally supplemented with chitin over 26 hours.**

|  | Growth data for cultures grown with chitin | | | | | |
| --- | --- | --- | --- | --- | --- | --- |
| Hours | Glucose + | | | Glucose - | | |
| 0 | 0.1575 | 0.1615 | 0.1118 | 0.1211 | 0.1449 | 0.1741 |
| 1 | 0.1496 | 0.0961 | 0.0799 | 0.0647 | 0.0561 | 0.0907 |
| 2 | 0.1449 | 0.1291 | 0.1822 | 0.0588 | 0.0929 | 0.1154 |
| 3 | 0.2462 | 0.2052 | 0.2009 | 0.1767 | 0.2042 | 0.1717 |
| 4 | 0.3283 | 0.3329 | 0.2458 | 0.2179 | 0.2088 | 0.1874 |
| 5 | 0.5092 | 0.4888 | 0.3712 | 0.3288 | 0.2858 | 0.3970 |
| 6 | 0.6751 | 0.6571 | 0.5918 | 0.4269 | 0.3504 | 0.3484 |
| 7 | 0.8175 | 0.9497 | 0.8101 | 0.4248 | 0.4796 | 0.5504 |
| 8 | 0.8645 | 0.9150 | 1.0670 | 0.5795 | 0.5868 | 0.5857 |
| 10 | 1.3820 | 1.5133 | 1.8972 | 1.0565 | 0.9630 | 0.9905 |
| 15 | 1.8943 | 1.8302 | 1.9718 | 1.1052 | 1.0437 | 1.0947 |
| 16 | 1.9315 | 2.0533 | 1.9430 | 1.0930 | 1.1036 | 1.0587 |
| 17 | 2.0163 | 2.1297 | 2.0971 | 1.1131 | 1.1184 | 1.1405 |
| 18 | 2.2246 | 2.1071 | 2.1271 | 1.1131 | 1.1568 | 1.1663 |
| 19 | 2.3083 | 2.0906 | 2.2109 | 1.1131 | 1.1568 | 1.1663 |
| 24 | 2.3316 | 2.1700 | 2.3391 | 1.1058 | 1.0833 | 1.2872 |
| 25 | 2.3573 | 2.3903 | 2.1931 | 1.1077 | 1.3891 | 1.1246 |
| 26 | 2.2136 | 2.3750 | 2.3890 | 1.0802 | 1.1683 |  |

**Table S5: OD_600_ values of *B. subtilis natto* growing in glucose rich and glucose poor medium additionally supplemented with scleroglucan over 26 hours.**

|  | Growth data for cultures grown with β-glucan | | | | | | | |
| --- | --- | --- | --- | --- | --- | --- | --- | --- |
| Hours | Glucose + | | | | Glucose - | | | |
| 0 | 0 | 0 | 0 | 0 | 0 | 0 | 0 | 0 |
| 1 | 0.0323 | 0.0854 | 0.1056 | 0.0258 | 0.0265 | 0.1349 | 0.0643 | 0.0407 |
| 2 | 0.0418 | 0.1196 | 0.1309 | 0.0595 | 0.0565 | 0.0498 | 0.1187 | 0.1792 |
| 3 | 0.08 | 0.1288 | 0.1532 | 0.0942 | 0.0613 | 0.1592 | 0.0838 | 0.124 |
| 4 | 0.0991 | 0.1431 | 0.1098 | 0.1265 | 0.06 | 0.1851 | 0.1113 | 0.0735 |
| 5 | 0.142 | 0.2694 | 0.1321 | 0.167 | 0.1211 | 0.1395 | 0.2237 | 0.1011 |
| 6 |  | 0.176 | 0.2081 | 0.2012 | 0.1344 | 0.1432 | 0.2154 | 0.0911 |
| 7 | 0.2191 | 0.1544 | 0.1533 | 0.177 | 0.1775 | 0.1817 | 0.1181 | 0.2646 |
| 8 | 0.2455 | 0.4012 | 0.1953 | 0.1851 | 0.1816 | 0.2048 | 0.1659 | 0.394 |
| 9 | 0.2191 | 0.2318 | 0.2046 |  | 0.1526 | 0.1806 | 0.1772 | 0.1946 |
| 10 | 0.3197 | 0.2492 | 0.2449 | 0.2836 | 0.2278 | 0.1754 | 0.2089 | 0.2461 |
| 13 | 1.4038 | 1.3905 | 1.4315 |  | 0.7876 | 0.7576 | 0.8479 |  |
| 14 | 1.4425 | 1.4403 | 1.437 |  | 0.8071 | 0.7717 | 0.9081 |  |
| 15 | 1.5217 | 1.5558 | 1.6373 |  | 0.9645 | 0.8606 | 0.9312 |  |
| 16 | 1.6646 | 1.5775 | 1.5721 |  | 0.8097 | 0.8326 | 0.8229 |  |
| 17 | 1.5607 | 1.6115 | 1.5412 |  | 0.8331 | 0.842 | 0.8265 |  |
| 18 | 1.6454 | 1.6094 | 1.5672 |  | 0.8217 | 0.8575 | 0.8114 |  |
| 19 | 1.6519 | 1.6204 | 1.6452 |  | 0.8204 | 0.8204 | 0.8573 |  |
| 20 | 1.6238 | 1.6637 | 1.6927 |  | 0.854 | 0.8196 | 0.8253 |  |
| 24 | 1.6885 | 1.7617 | 1.787 | 1.8365 | 1.1206 | 0.9069 | 1.0441 | 1.206 |
| 25 | 1.7276 | 1.5963 | 1.6732 | 1.6001 | 0.949 | 0.961 | 0.9637 | 0.928 |
| 26 | 1.758 | 1.6669 | 1.6689 | 1.7286 | 0.9889 | 0.9526 | 0.9545 | 1.0181 |

**Table S6: OD_600_ values of *B. subtilis natto* growing in glucose rich and glucose poor medium additionally supplemented with peptone over 26 hours.**

|  | Growth data for cultures grown with peptone | | | | | |
| --- | --- | --- | --- | --- | --- | --- |
| Hours | Glucose + | | | Glucose - | | |
| 0 | 0.0284 | 0.026 | 0.0298 | 0.0287 | 0.0274 | 0.0312 |
| 1 | 0.0443 | 0.0471 | 0.044 | 0.0454 | 0.0471 | 0.0494 |
| 2 | 0.0964 | 0.095 | 0.0996 | 0.0974 | 0.1104 | 0.1073 |
| 3 | 0.2494 | 0.2827 | 0.2858 | 0.2885 | 0.2634 | 0.2947 |
| 4 | 0.624 | 0.3087 | 0.5543 | 0.5536 | 0.6062 | 0.6176 |
| 6 | 1.6885 | 1.7204 | 1.8031 | 1.4131 | 1.4531 | 1.2831 |
| 7 | 2.0485 | 1.982 | 2.1061 | 1.685 | 1.7631 | 1.7849 |
| 8 | 2.1948 | 2.137 | 2.2195 | 1.9077 | 1.8921 | 1.7871 |
| 9 | 2.197 | 2.1855 | 2.2746 | 1.9318 | 1.9247 | 1.813 |
| 15 | 2.0811 | 2.1495 | 2.0389 | 1.4536 | 1.1495 | 2.0389 |
| 16 | 2.0437 | 2.0611 | 2.0248 | 1.4094 | 1.3919 | 0 |
| 17 | 2.0345 | 2.0315 | 2.0129 | 1.4011 | 1.4105 | 1.3844 |
| 24 | 2.2289 | 2.3834 | 2.2444 | 1.9369 | 1.8409 | 1.6636 |
| 25 | 2.2831 | 2.2507 | 2.4434 | 1.8268 | 1.8405 | 1.6533 |
| 26 | 2.2491 | 2.4334 | 2.2527 | 1.8268 | 1.8405 | 1.6371 |

**Table S7: MS-identified secreted proteins of *B. subtilis natto* in supernatant (MASCOT database search results/NCBI database)*.***

|  | ID | Protein | Mol wt (Da) | Score | Matches | Sequences | emPAI |
| --- | --- | --- | --- | --- | --- | --- | --- |
| BAND 1 | [gi\|489325659](http://bio-mascot1.chem.umu.se/mascot/cgi/protein_view.pl?file=..%2Fdata%2F20190509%2FF115973.dat&hit=gi%7C489325659&db_idx=1&px=1&ave_thresh=32&report=0&_sigthreshold=0.05&_msresflags=1089&_msresflags2=256&percolate=-1&percolate_rt=0&_minpeplen=7&sessionID=Vaibhav_S_2854232444264669) | Peptide-binding protein | 61923 | 1687 | 39(39) | 18(18) | 1.70 |
|  | [gi\|489325005](http://bio-mascot1.chem.umu.se/mascot/cgi/protein_view.pl?file=..%2Fdata%2F20190509%2FF115973.dat&hit=gi%7C489325005&db_idx=1&px=1&ave_thresh=32&report=0&_sigthreshold=0.05&_msresflags=1089&_msresflags2=256&percolate=-1&percolate_rt=0&_minpeplen=7&sessionID=Vaibhav_S_2854232444264669) | Dihydrolipoyl dehydrogenase | 49834 | 646 | 16(16) | 12(12) | 1.27 |
|  | [gi\|740642079](http://bio-mascot1.chem.umu.se/mascot/cgi/protein_view.pl?file=..%2Fdata%2F20190509%2FF115973.dat&hit=gi%7C740642079&db_idx=1&px=1&ave_thresh=32&report=0&_sigthreshold=0.05&_msresflags=1089&_msresflags2=256&percolate=-1&percolate_rt=0&_minpeplen=7&sessionID=Vaibhav_S_2854232444264669) | Peptidase G2 | 85032 | 433 | 9(9) | 6(6) |  |
|  | [gi\|489321409](http://bio-mascot1.chem.umu.se/mascot/cgi/protein_view.pl?file=..%2Fdata%2F20190509%2FF115973.dat&hit=gi%7C489321409&db_idx=1&px=1&ave_thresh=32&report=0&_sigthreshold=0.05&_msresflags=1089&_msresflags2=256&percolate=-1&percolate_rt=0&_minpeplen=7&sessionID=Vaibhav_S_2854232444264669) | Cytosol aminopeptidase | 53844 | 177 | 4(4) | 4(4) | 0.29 |
| BAND 3 | [gi\|449027635](http://bio-mascot1.chem.umu.se/mascot/cgi/protein_view.pl?file=..%2Fdata%2F20190509%2FF115975.dat&hit=gi%7C449027635&db_idx=1&px=1&ave_thresh=33&report=0&_sigthreshold=0.05&_msresflags=1089&_msresflags2=256&percolate=-1&percolate_rt=0&_minpeplen=7&sessionID=Vaibhav_S_3950297279209413) | Peptidase M42 | 38009 | 276 | 10(10) | 8(8) | 1.04 |
|  | [gi\|1498320](http://bio-mascot1.chem.umu.se/mascot/cgi/protein_view.pl?file=..%2Fdata%2F20190509%2FF115975.dat&hit=gi%7C1498320&db_idx=1&px=1&ave_thresh=33&_ignoreionsscorebelow=-1&report=0&_sigthreshold=0.05&_msresflags=1097&_msresflags2=256&percolate=-1&percolate_rt=0&_minpeplen=7&sessionID=Vaibhav_S_3950297279209413) | Cell wall-associated protease precursor/Peptidase S8 | 96444 | 276 | 6(6) | 6(6) | 0.24 |
|  | [gi\|518544361](http://bio-mascot1.chem.umu.se/mascot/cgi/protein_view.pl?file=..%2Fdata%2F20190509%2FF115975.dat&hit=gi%7C518544361&db_idx=1&px=1&ave_thresh=33&_ignoreionsscorebelow=-1&report=0&_sigthreshold=0.05&_msresflags=1097&_msresflags2=256&percolate=-1&percolate_rt=0&_minpeplen=7&sessionID=Vaibhav_S_3950297279209413) | Gamma-glutamyltransferase | 64062 | 137 | 2(2) | 2(2) | 0.11 |
|  | [gi\|142609](http://bio-mascot1.chem.umu.se/mascot/cgi/protein_view.pl?file=..%2Fdata%2F20190509%2FF115975.dat&hit=gi%7C142609&db_idx=1&px=1&ave_thresh=33&_ignoreionsscorebelow=-1&report=0&_sigthreshold=0.05&_msresflags=1097&_msresflags2=256&percolate=-1&percolate_rt=0&_minpeplen=7&sessionID=Vaibhav_S_3950297279209413) | Bacillopeptidase F | 91762 | 130 | 4 (4) | 2 (2) | 0.08 |
|  | [gi\|489246357](http://bio-mascot1.chem.umu.se/mascot/cgi/protein_view.pl?file=..%2Fdata%2F20190509%2FF115975.dat&hit=gi%7C489246357&db_idx=1&px=1&ave_thresh=33&_ignoreionsscorebelow=-1&report=0&_sigthreshold=0.05&_msresflags=1097&_msresflags2=256&percolate=-1&percolate_rt=0&_minpeplen=7&sessionID=Vaibhav_S_3950297279209413) | Dihydrolipoyl dehydrogenase | 49765 | 113 | 2 (2) | 2 (2) | 0.15 |
| BAND 5 | [gi\|489315046](http://bio-mascot1.chem.umu.se/mascot/cgi/protein_view.pl?file=..%2Fdata%2F20190509%2FF115977.dat&hit=gi%7C489315046&db_idx=1&px=1&ave_thresh=33&_ignoreionsscorebelow=-1&report=0&_sigthreshold=0.05&_msresflags=1097&_msresflags2=256&percolate=-1&percolate_rt=0&_minpeplen=7&sessionID=Vaibhav_S_3950297279209413) | Peptidase M28 | 39279 | 459 | 12(12) | 8(8) | 0.99 |
|  | [gi\|1498320](http://bio-mascot1.chem.umu.se/mascot/cgi/protein_view.pl?file=..%2Fdata%2F20190509%2FF115977.dat&hit=gi%7C1498320&db_idx=1&px=1&ave_thresh=33&_ignoreionsscorebelow=-1&report=0&_sigthreshold=0.05&_msresflags=1097&_msresflags2=256&percolate=-1&percolate_rt=0&_minpeplen=7&sessionID=Vaibhav_S_3950297279209413) | Cell wall-associated protease precursor | 96444 | 378 | 9(9) | 9(9) | 0.38 |
|  | [gi\|351470036](http://bio-mascot1.chem.umu.se/mascot/cgi/protein_view.pl?file=..%2Fdata%2F20190509%2FF115977.dat&hit=gi%7C351470036&db_idx=1&px=1&ave_thresh=33&_ignoreionsscorebelow=-1&report=0&_sigthreshold=0.05&_msresflags=1097&_msresflags2=256&percolate=-1&percolate_rt=0&_minpeplen=7&sessionID=Vaibhav_S_3950297279209413) | Aminopeptidase | 38040 | 300 | 10(10) | 8(8) | 1.04 |
|  | [gi\|62511911](http://bio-mascot1.chem.umu.se/mascot/cgi/protein_view.pl?file=..%2Fdata%2F20190509%2FF115977.dat&hit=gi%7C62511911&db_idx=1&px=1&ave_thresh=33&_ignoreionsscorebelow=-1&report=0&_sigthreshold=0.05&_msresflags=1097&_msresflags2=256&percolate=-1&percolate_rt=0&_minpeplen=7&sessionID=Vaibhav_S_3950297279209413) | Extracellular protease vpr, partial | 68092 | 286 | 5(5) | 5(5) | 0.29 |
|  | [gi\|489314908](http://bio-mascot1.chem.umu.se/mascot/cgi/protein_view.pl?file=..%2Fdata%2F20190509%2FF115977.dat&hit=gi%7C489314908&db_idx=1&px=1&ave_thresh=33&_ignoreionsscorebelow=-1&report=0&_sigthreshold=0.05&_msresflags=1097&_msresflags2=256&percolate=-1&percolate_rt=0&_minpeplen=7&sessionID=Vaibhav_S_3950297279209413) | Dihydrolipoyl dehydrogenase | 49820 | 273 | 7(7) | 6(6) | 0.51 |
|  | [gi\|504289878](http://bio-mascot1.chem.umu.se/mascot/cgi/protein_view.pl?file=..%2Fdata%2F20190509%2FF115977.dat&hit=gi%7C504289878&db_idx=1&px=1&ave_thresh=33&_ignoreionsscorebelow=-1&report=0&_sigthreshold=0.05&_msresflags=1097&_msresflags2=256&percolate=-1&percolate_rt=0&_minpeplen=7&sessionID=Vaibhav_S_3950297279209413) | Gamma-glutamyltransferase | 64171 | 188 | 3(3) | 3(3) | 0.17 |
|  | [gi\|757753486](http://bio-mascot1.chem.umu.se/mascot/cgi/protein_view.pl?file=..%2Fdata%2F20190509%2FF115977.dat&hit=gi%7C757753486&db_idx=1&px=1&ave_thresh=33&_ignoreionsscorebelow=-1&report=0&_sigthreshold=0.05&_msresflags=1097&_msresflags2=256&percolate=-1&percolate_rt=0&_minpeplen=7&sessionID=Vaibhav_S_3950297279209413) | Major capsid protein | 41984 | 183 | 2(2) | 2(2) | 0.18 |
|  | [gi\|757753483](http://bio-mascot1.chem.umu.se/mascot/cgi/protein_view.pl?file=..%2Fdata%2F20190509%2FF115977.dat&hit=gi%7C757753483&db_idx=1&px=1&ave_thresh=33&_ignoreionsscorebelow=-1&report=0&_sigthreshold=0.05&_msresflags=1097&_msresflags2=256&percolate=-1&percolate_rt=0&_minpeplen=7&sessionID=Vaibhav_S_3950297279209413) | Hypothetical protein | 16579 | 109 | 2(2) | 2(2) | 0.49 |
| BAND 7 | [gi\|489311961](http://bio-mascot1.chem.umu.se/mascot/cgi/protein_view.pl?file=..%2Fdata%2F20190509%2FF115979.dat&hit=gi%7C489311961&db_idx=1&px=1&ave_thresh=32&report=0&_sigthreshold=0.05&_msresflags=1089&_msresflags2=256&percolate=-1&percolate_rt=0&_minpeplen=7&sessionID=Vaibhav_S_2854232444264669) | DNA starvation/stationary phase protection protein | 16598 | 324 | 4 (4) | 2(2) | 0.49 |
|  | [gi\|1303851](http://bio-mascot1.chem.umu.se/mascot/cgi/protein_view.pl?file=..%2Fdata%2F20190509%2FF115979.dat&hit=gi%7C1303851&db_idx=1&px=1&ave_thresh=32&report=0&_sigthreshold=0.05&_msresflags=1089&_msresflags2=256&percolate=-1&percolate_rt=0&_minpeplen=7&sessionID=Vaibhav_S_2854232444264669) | Superoxide dismutase | 25319 | 196 | 3(3) | 2(2) | 0.31 |
|  | [gi\|1498320](http://bio-mascot1.chem.umu.se/mascot/cgi/protein_view.pl?file=..%2Fdata%2F20190509%2FF115979.dat&hit=gi%7C1498320&db_idx=1&px=1&ave_thresh=32&report=0&_sigthreshold=0.05&_msresflags=1089&_msresflags2=256&percolate=-1&percolate_rt=0&_minpeplen=7&sessionID=Vaibhav_S_2854232444264669) | Cell wall-associated protease precursor/Peptidase S8 | 96444 | 117 | 2(2) | 2(2) | 0.07 |
|  | [gi\|402483032](http://bio-mascot1.chem.umu.se/mascot/cgi/protein_view.pl?file=..%2Fdata%2F20190509%2FF115979.dat&hit=gi%7C402483032&db_idx=1&px=1&ave_thresh=32&report=0&_sigthreshold=0.05&_msresflags=1089&_msresflags2=256&percolate=-1&percolate_rt=0&_minpeplen=7&sessionID=Vaibhav_S_2854232444264669) | Serine hydroxymethyltransferase | 45680 | 95 | 2(2) | 2(2) | 0.16 |
|  | [gi\|52782799](http://bio-mascot1.chem.umu.se/mascot/cgi/protein_view.pl?file=..%2Fdata%2F20190509%2FF115979.dat&hit=gi%7C52782799&db_idx=1&px=1&ave_thresh=32&report=0&_sigthreshold=0.05&_msresflags=1089&_msresflags2=256&percolate=-1&percolate_rt=0&_minpeplen=7&sessionID=Vaibhav_S_2854232444264669) | Gamma-glutamyltranspeptidase, Glutathione Hydrolase | 64074 | 91 | 2(2) | 2(2) | 0.11 |
| BAND 8 | [gi\|489311961](http://bio-mascot1.chem.umu.se/mascot/cgi/protein_view.pl?file=..%2Fdata%2F20190509%2FF115980.dat&hit=gi%7C489311961&db_idx=1&px=1&ave_thresh=33&report=0&_sigthreshold=0.05&_msresflags=1089&_msresflags2=256&percolate=-1&percolate_rt=0&_minpeplen=7&sessionID=Vaibhav_S_2854232444264669) | DNA starvation/stationary phase protection protein | 16598 | 364 | 7(7) | 6(6) | 2.3 |
|  | [gi\|1303851](http://bio-mascot1.chem.umu.se/mascot/cgi/protein_view.pl?file=..%2Fdata%2F20190509%2FF115980.dat&hit=gi%7C1303851&db_idx=1&px=1&ave_thresh=33&report=0&_sigthreshold=0.05&_msresflags=1089&_msresflags2=256&percolate=-1&percolate_rt=0&_minpeplen=7&sessionID=Vaibhav_S_2854232444264669) | Superoxide dismutase | 25319 | 215 | 3(3) | 3(3) | 0.49 |
|  | [gi\|402483032](http://bio-mascot1.chem.umu.se/mascot/cgi/protein_view.pl?file=..%2Fdata%2F20190509%2FF115980.dat&hit=gi%7C402483032&db_idx=1&px=1&ave_thresh=33&report=0&_sigthreshold=0.05&_msresflags=1089&_msresflags2=256&percolate=-1&percolate_rt=0&_minpeplen=7&sessionID=Vaibhav_S_2854232444264669) | Serine hydroxymethyltransferase | 45680 | 133 | 3(3) | 3(3) | 0.25 |
|  | [gi\|489324264](http://bio-mascot1.chem.umu.se/mascot/cgi/protein_view.pl?file=..%2Fdata%2F20190509%2FF115980.dat&hit=gi%7C489324264&db_idx=1&px=1&ave_thresh=33&report=0&_sigthreshold=0.05&_msresflags=1089&_msresflags2=256&percolate=-1&percolate_rt=0&_minpeplen=7&sessionID=Vaibhav_S_2854232444264669) | Aconitate hydratase 1 | 99523 | 471 | 11(11) | 11(11) | 0.46 |
|  | [gi\|351472723](http://bio-mascot1.chem.umu.se/mascot/cgi/protein_view.pl?file=..%2Fdata%2F20190509%2FF115980.dat&hit=gi%7C351472723&db_idx=1&px=1&ave_thresh=33&report=0&_sigthreshold=0.05&_msresflags=1089&_msresflags2=256&percolate=-1&percolate_rt=0&_minpeplen=7&sessionID=Vaibhav_S_2854232444264669) | Glucose-6-phosphate isomerase | 51072 | 380 | 8(8) | 6(6) | 0.49 |
|  | [gi\|259090092](http://bio-mascot1.chem.umu.se/mascot/cgi/protein_view.pl?file=..%2Fdata%2F20190509%2FF115980.dat&hit=gi%7C259090092&db_idx=1&px=1&ave_thresh=33&report=0&_sigthreshold=0.05&_msresflags=1089&_msresflags2=256&percolate=-1&percolate_rt=0&_minpeplen=7&sessionID=Vaibhav_S_2854232444264669) | Triscatecholate Siderophore Binding Protein | 34669 | 311 | 9(9) | 7(7) | 0.98 |
|  | [gi\|489311187](http://bio-mascot1.chem.umu.se/mascot/cgi/protein_view.pl?file=..%2Fdata%2F20190509%2FF115980.dat&hit=gi%7C489311187&db_idx=1&px=1&ave_thresh=33&report=0&_sigthreshold=0.05&_msresflags=1089&_msresflags2=256&percolate=-1&percolate_rt=0&_minpeplen=7&sessionID=Vaibhav_S_2854232444264669) | Acireductone dioxygenase | 20713 | 244 | 6(6) | 5(5) | 1.24 |
|  | [gi\|489313200](http://bio-mascot1.chem.umu.se/mascot/cgi/protein_view.pl?file=..%2Fdata%2F20190509%2FF115980.dat&hit=gi%7C489313200&db_idx=1&px=1&ave_thresh=33&report=0&_sigthreshold=0.05&_msresflags=1089&_msresflags2=256&percolate=-1&percolate_rt=0&_minpeplen=7&sessionID=Vaibhav_S_2854232444264669) | Phage-like element PBSX protein XkdM | 16345 | 191 | 5(5) | 5(5) | 1.76 |
|  | [gi\|489317245](http://bio-mascot1.chem.umu.se/mascot/cgi/protein_view.pl?file=..%2Fdata%2F20190509%2FF115980.dat&hit=gi%7C489317245&db_idx=1&px=1&ave_thresh=33&report=0&_sigthreshold=0.05&_msresflags=1089&_msresflags2=256&percolate=-1&percolate_rt=0&_minpeplen=7&sessionID=Vaibhav_S_2854232444264669) | Oligoendopeptidase F | 77080 | 133 | 2(2) | 2(2) | 0.09 |
|  | [gi\|6729823](http://bio-mascot1.chem.umu.se/mascot/cgi/protein_view.pl?file=..%2Fdata%2F20190509%2FF115980.dat&hit=gi%7C6729823&db_idx=1&px=1&ave_thresh=33&report=0&_sigthreshold=0.05&_msresflags=1089&_msresflags2=256&percolate=-1&percolate_rt=0&_minpeplen=7&sessionID=Vaibhav_S_2854232444264669) | Pectate Lyase | 45486 | 129 | 2(2) | 2(2) | 0.16 |

**Table S8: MS-identified *A. bisporus* proteins from BFC and *B. subtilis natto* supernatant (MASCOT database search results)*.***

|  | ID | Protein | kDa | Score | Matches | Sequences | emPAI |
| --- | --- | --- | --- | --- | --- | --- | --- |
| BAND 3 | [gi\|659835403](http://bio-mascot1.chem.umu.se/mascot/cgi/protein_view.pl?file=..%2Fdata%2F20190509%2FF116019.dat&hit=gi%7C659835403&db_idx=1&px=1&ave_thresh=50&report=0&_sigthreshold=0.05&_msresflags=1025&_msresflags2=256&percolate=-1&percolate_rt=0&_minpeplen=7&sessionID=Vaibhav_S_3950297279209413) | Tyrosinase Chain A | 43,675 | 486 | 8(8) | 7(7) | 0.73 |
|  | [gi\|568455239](http://bio-mascot1.chem.umu.se/mascot/cgi/protein_view.pl?file=..%2Fdata%2F20190509%2FF116019.dat&hit=gi%7C568455239&db_idx=1&px=1&ave_thresh=50&_ignoreionsscorebelow=-1&report=0&_sigthreshold=0.05&_msresflags=1033&_msresflags2=256&percolate=-1&percolate_rt=0&_minpeplen=7&sessionID=Vaibhav_S_3950297279209413) | AGABI2DRAFT_192455 | 61,398 | 176 | 2(2) | 2(2) | 0.12 |
| BAND 4 | [gi\|568447153](http://bio-mascot1.chem.umu.se/mascot/cgi/protein_view.pl?file=..%2Fdata%2F20190509%2FF116020.dat&hit=gi%7C568447153&db_idx=1&px=1&ave_thresh=50&_ignoreionsscorebelow=-1&report=0&_sigthreshold=0.05&_msresflags=1033&_msresflags2=256&percolate=-1&percolate_rt=0&_minpeplen=7&sessionID=Vaibhav_S_3950297279209413) | Putative heat shock hsp70 protein | 71,212 | 1010 | 19(19) | 11(11) | 0.69 |
|  | [gi\|568434474](http://bio-mascot1.chem.umu.se/mascot/cgi/protein_view.pl?file=..%2Fdata%2F20190509%2FF116020.dat&hit=gi%7C568434474&db_idx=1&px=1&ave_thresh=50&_ignoreionsscorebelow=-1&report=0&_sigthreshold=0.05&_msresflags=1033&_msresflags2=256&percolate=-1&percolate_rt=0&_minpeplen=7&sessionID=Vaibhav_S_3950297279209413) | AGABI2DRAFT_189297 | 47,546 | 815 | 11(11) | 9(9) | 0.89 |
|  | [gi\|597963485](http://bio-mascot1.chem.umu.se/mascot/cgi/protein_view.pl?file=..%2Fdata%2F20190509%2FF116020.dat&hit=gi%7C597963485&db_idx=1&px=1&ave_thresh=50&_ignoreionsscorebelow=-1&report=0&_sigthreshold=0.05&_msresflags=1033&_msresflags2=256&percolate=-1&percolate_rt=0&_minpeplen=7&sessionID=Vaibhav_S_3950297279209413) | AGABI1DRAFT_32539 | 44,299 | 772 | 13(13) | 9(9) | 0.98 |
|  | [gi\|568448279](http://bio-mascot1.chem.umu.se/mascot/cgi/protein_view.pl?file=..%2Fdata%2F20190509%2FF116020.dat&hit=gi%7C568448279&db_idx=1&px=1&ave_thresh=50&_ignoreionsscorebelow=-1&report=0&_sigthreshold=0.05&_msresflags=1033&_msresflags2=256&percolate=-1&percolate_rt=0&_minpeplen=7&sessionID=Vaibhav_S_3950297279209413) | AGABI2DRAFT_141355 | 51,472 | 660 | 8(8) | 8(8) | 0.69 |
|  | [gi\|568456924](http://bio-mascot1.chem.umu.se/mascot/cgi/protein_view.pl?file=..%2Fdata%2F20190509%2FF116020.dat&hit=gi%7C568456924&db_idx=1&px=1&ave_thresh=50&_ignoreionsscorebelow=-1&report=0&_sigthreshold=0.05&_msresflags=1033&_msresflags2=256&percolate=-1&percolate_rt=0&_minpeplen=7&sessionID=Vaibhav_S_3950297279209413) | AGABI2DRAFT_192925 | 50,087 | 611 | 8(8) | 7(7) | 0.6 |
|  | [gi\|339717368](http://bio-mascot1.chem.umu.se/mascot/cgi/protein_view.pl?file=..%2Fdata%2F20190509%2FF116020.dat&hit=gi%7C339717368&db_idx=1&px=1&ave_thresh=50&_ignoreionsscorebelow=-1&report=0&_sigthreshold=0.05&_msresflags=1033&_msresflags2=256&percolate=-1&percolate_rt=0&_minpeplen=7&sessionID=Vaibhav_S_3950297279209413) | Tyrosinase (abppo4) Chain A | 45,330 | 573 | 8(8) | 6(6) | 0.56 |
|  | [gi\|597984767](http://bio-mascot1.chem.umu.se/mascot/cgi/protein_view.pl?file=..%2Fdata%2F20190509%2FF116020.dat&hit=gi%7C597984767&db_idx=1&px=1&ave_thresh=50&_ignoreionsscorebelow=-1&report=0&_sigthreshold=0.05&_msresflags=1033&_msresflags2=256&percolate=-1&percolate_rt=0&_minpeplen=7&sessionID=Vaibhav_S_265890183135387) | AGABI1DRAFT_114468 | 68,408 | 569 | 11(11) | 8(8) | 0.49 |
|  | [gi\|597985539](http://bio-mascot1.chem.umu.se/mascot/cgi/protein_view.pl?file=..%2Fdata%2F20190509%2FF116020.dat&hit=gi%7C597985539&db_idx=1&px=1&ave_thresh=50&_ignoreionsscorebelow=-1&report=0&_sigthreshold=0.05&_msresflags=1033&_msresflags2=256&percolate=-1&percolate_rt=0&_minpeplen=7&sessionID=Vaibhav_S_265890183135387) | AGABI1DRAFT_75994 | 48,118 | 523 | 7(7) | 6(6) | 0.52 |
|  | [gi\|568453554](http://bio-mascot1.chem.umu.se/mascot/cgi/protein_view.pl?file=..%2Fdata%2F20190509%2FF116020.dat&hit=gi%7C568453554&db_idx=1&px=1&ave_thresh=50&_ignoreionsscorebelow=-1&report=0&_sigthreshold=0.05&_msresflags=1033&_msresflags2=256&percolate=-1&percolate_rt=0&_minpeplen=7&sessionID=Vaibhav_S_265890183135387) | AGABI2DRAFT_192052 | 45,096 | 415 | 4(4) | 4(4) | 0.35 |
|  | [gi\|568440122](http://bio-mascot1.chem.umu.se/mascot/cgi/protein_view.pl?file=..%2Fdata%2F20190509%2FF116020.dat&hit=gi%7C568440122&db_idx=1&px=1&ave_thresh=50&_ignoreionsscorebelow=-1&report=0&_sigthreshold=0.05&_msresflags=1033&_msresflags2=256&percolate=-1&percolate_rt=0&_minpeplen=7&sessionID=Vaibhav_S_265890183135387) | Heat shock hsp70 protein | 73,504 | 390 | 4(4) | 4(4) | 0.2 |
|  | [gi\|568442104](http://bio-mascot1.chem.umu.se/mascot/cgi/protein_view.pl?file=..%2Fdata%2F20190509%2FF116020.dat&hit=gi%7C568442104&db_idx=1&px=1&ave_thresh=50&_ignoreionsscorebelow=-1&report=0&_sigthreshold=0.05&_msresflags=1033&_msresflags2=256&percolate=-1&percolate_rt=0&_minpeplen=7&sessionID=Vaibhav_S_265890183135387) | AGABI2DRAFT_139240 | 46,464 | 341 | 4(4) | 4(4) | 0.34 |
|  | [gi\|568464341](http://bio-mascot1.chem.umu.se/mascot/cgi/protein_view.pl?file=..%2Fdata%2F20190509%2FF116020.dat&hit=gi%7C568464341&db_idx=1&px=1&ave_thresh=50&_ignoreionsscorebelow=-1&report=0&_sigthreshold=0.05&_msresflags=1033&_msresflags2=256&percolate=-1&percolate_rt=0&_minpeplen=7&sessionID=Vaibhav_S_265890183135387) | AGABI2DRAFT_138150 | 47,486 | 334 | 4(4) | 4(4) | 0.33 |
|  | [gi\|597986243](http://bio-mascot1.chem.umu.se/mascot/cgi/protein_view.pl?file=..%2Fdata%2F20190509%2FF116020.dat&hit=gi%7C597986243&db_idx=1&px=1&ave_thresh=50&_ignoreionsscorebelow=-1&report=0&_sigthreshold=0.05&_msresflags=1033&_msresflags2=256&percolate=-1&percolate_rt=0&_minpeplen=7&sessionID=Vaibhav_S_265890183135387) | AGABI1DRAFT_114685 | 48,352 | 311 | 4(4) | 4(4) | 0.32 |
|  | [gi\|568451647](http://bio-mascot1.chem.umu.se/mascot/cgi/protein_view.pl?file=..%2Fdata%2F20190509%2FF116020.dat&hit=gi%7C568451647&db_idx=1&px=1&ave_thresh=50&_ignoreionsscorebelow=-1&report=0&_sigthreshold=0.05&_msresflags=1033&_msresflags2=256&percolate=-1&percolate_rt=0&_minpeplen=7&sessionID=Vaibhav_S_265890183135387) | AGABI2DRAFT_191563 | 45,796 | 286 | 3(3) | 3(3) | 0.25 |
|  | [gi\|568458572](http://bio-mascot1.chem.umu.se/mascot/cgi/protein_view.pl?file=..%2Fdata%2F20190509%2FF116020.dat&hit=gi%7C568458572&db_idx=1&px=1&ave_thresh=50&_ignoreionsscorebelow=-1&report=0&_sigthreshold=0.05&_msresflags=1033&_msresflags2=256&percolate=-1&percolate_rt=0&_minpeplen=7&sessionID=Vaibhav_S_265890183135387) | AGABI2DRAFT_193317 | 59788 | 208 | 4(4) | 3(3) | 0.18 |
|  | [gi\|568435000](http://bio-mascot1.chem.umu.se/mascot/cgi/protein_view.pl?file=..%2Fdata%2F20190509%2FF116020.dat&hit=gi%7C568435000&db_idx=1&px=1&ave_thresh=50&_ignoreionsscorebelow=-1&report=0&_sigthreshold=0.05&_msresflags=1033&_msresflags2=256&percolate=-1&percolate_rt=0&_minpeplen=7&sessionID=Vaibhav_S_265890183135387) | AGABI2DRAFT_189449 | 48,494 | 199 | 2(2) | 2(2) | 0.15 |
|  | [gi\|568447001](http://bio-mascot1.chem.umu.se/mascot/cgi/protein_view.pl?file=..%2Fdata%2F20190509%2FF116020.dat&hit=gi%7C568447001&db_idx=1&px=1&ave_thresh=50&_ignoreionsscorebelow=-1&report=0&_sigthreshold=0.05&_msresflags=1033&_msresflags2=256&percolate=-1&percolate_rt=0&_minpeplen=7&sessionID=Vaibhav_S_265890183135387) | AGABI2DRAFT_64000 | 133,234 | 170 | 2(2) | 2(2) | 0.05 |
|  | [gi\|568444913](http://bio-mascot1.chem.umu.se/mascot/cgi/protein_view.pl?file=..%2Fdata%2F20190509%2FF116020.dat&hit=gi%7C568444913&db_idx=1&px=1&ave_thresh=50&_ignoreionsscorebelow=-1&report=0&_sigthreshold=0.05&_msresflags=1033&_msresflags2=256&percolate=-1&percolate_rt=0&_minpeplen=7&sessionID=Vaibhav_S_265890183135387) | AGABI2DRAFT_196132 | 54,893 | 151 | 2(2) | 2(2) | 0.13 |
|  | [gi\|6821991](http://bio-mascot1.chem.umu.se/mascot/cgi/protein_view.pl?file=..%2Fdata%2F20190509%2FF116020.dat&hit=gi%7C6821991&db_idx=1&px=1&ave_thresh=50&_ignoreionsscorebelow=-1&report=0&_sigthreshold=0.05&_msresflags=1033&_msresflags2=256&percolate=-1&percolate_rt=0&_minpeplen=7&sessionID=Vaibhav_S_265890183135387) | Glucuronyl hydrolase, partial | 44,022 | 145 | 2(2) | 2(2) | 0.17 |
|  | [gi\|568443924](http://bio-mascot1.chem.umu.se/mascot/cgi/protein_view.pl?file=..%2Fdata%2F20190509%2FF116020.dat&hit=gi%7C568443924&db_idx=1&px=1&ave_thresh=50&_ignoreionsscorebelow=-1&report=0&_sigthreshold=0.05&_msresflags=1033&_msresflags2=256&percolate=-1&percolate_rt=0&_minpeplen=7&sessionID=Vaibhav_S_265890183135387) | AGABI2DRAFT_229390 | 54,997 | 145 | 2(2) | 2(2) | 0.13 |
|  | [gi\|568433853](http://bio-mascot1.chem.umu.se/mascot/cgi/protein_view.pl?file=..%2Fdata%2F20190509%2FF116020.dat&hit=gi%7C568433853&db_idx=1&px=1&ave_thresh=50&_ignoreionsscorebelow=-1&report=0&_sigthreshold=0.05&_msresflags=1033&_msresflags2=256&percolate=-1&percolate_rt=0&_minpeplen=7&sessionID=Vaibhav_S_265890183135387) | AGABI2DRAFT_189140 | 58,792 | 115 | 2(2) | 2(2) | 0.12 |
| BAND 5 | [gi\|659835403](http://bio-mascot1.chem.umu.se/mascot/cgi/protein_view.pl?file=..%2Fdata%2F20190509%2FF116021.dat&hit=gi%7C659835403&db_idx=1&px=1&ave_thresh=50&_ignoreionsscorebelow=-1&report=0&_sigthreshold=0.05&_msresflags=1033&_msresflags2=256&percolate=-1&percolate_rt=0&_minpeplen=7&sessionID=Vaibhav_S_3950297279209413) | Tyrosinase (abppo4) Chain A | 43,675 | 428 | 7(7) | 6(6) | 0.6 |
|  | [gi\|597985539](http://bio-mascot1.chem.umu.se/mascot/cgi/protein_view.pl?file=..%2Fdata%2F20190509%2FF116021.dat&hit=gi%7C597985539&db_idx=1&px=1&ave_thresh=50&_ignoreionsscorebelow=-1&report=0&_sigthreshold=0.05&_msresflags=1033&_msresflags2=256&percolate=-1&percolate_rt=0&_minpeplen=7&sessionID=Vaibhav_S_3950297279209413) | AGABI1DRAFT_75994 | 48,118 | 269 | 3(3) | 3(3) | 0.24 |
|  | [gi\|568448567](http://bio-mascot1.chem.umu.se/mascot/cgi/protein_view.pl?file=..%2Fdata%2F20190509%2FF116021.dat&hit=gi%7C568448567&db_idx=1&px=1&ave_thresh=50&_ignoreionsscorebelow=-1&report=0&_sigthreshold=0.05&_msresflags=1033&_msresflags2=256&percolate=-1&percolate_rt=0&_minpeplen=7&sessionID=Vaibhav_S_3950297279209413) | AGABI2DRAFT_63774 | 60,496 | 209 | 3(3) | 3(3) | 0.18 |
|  | [gi\|568464341](http://bio-mascot1.chem.umu.se/mascot/cgi/protein_view.pl?file=..%2Fdata%2F20190509%2FF116021.dat&hit=gi%7C568464341&db_idx=1&px=1&ave_thresh=50&_ignoreionsscorebelow=-1&report=0&_sigthreshold=0.05&_msresflags=1033&_msresflags2=256&percolate=-1&percolate_rt=0&_minpeplen=7&sessionID=Vaibhav_S_3950297279209413) | AGABI2DRAFT_138150 | 47,486 | 116 | 2(2) | 2(2) | 0.15 |
| BAND 6 | [gi\|568448279](http://bio-mascot1.chem.umu.se/mascot/cgi/protein_view.pl?file=..%2Fdata%2F20190509%2FF116022.dat&hit=gi%7C568448279&db_idx=1&px=1&ave_thresh=50&_ignoreionsscorebelow=-1&report=0&_sigthreshold=0.05&_msresflags=1033&_msresflags2=256&percolate=-1&percolate_rt=0&_minpeplen=7&sessionID=Vaibhav_S_2095245552053707) | AGABI2DRAFT_141355 | 51,472 | 1535 | 20(20) | 18(18) | 2.28 |
|  | [gi\|568443924](http://bio-mascot1.chem.umu.se/mascot/cgi/protein_view.pl?file=..%2Fdata%2F20190509%2FF116022.dat&hit=gi%7C568443924&db_idx=1&px=1&ave_thresh=50&_ignoreionsscorebelow=-1&report=0&_sigthreshold=0.05&_msresflags=1033&_msresflags2=256&percolate=-1&percolate_rt=0&_minpeplen=7&sessionID=Vaibhav_S_2095245552053707) | AGABI2DRAFT_229390 | 54,997 | 1108 | 16(16) | 13(13) | 1.23 |
|  | [gi\|568456924](http://bio-mascot1.chem.umu.se/mascot/cgi/protein_view.pl?file=..%2Fdata%2F20190509%2FF116022.dat&hit=gi%7C568456924&db_idx=1&px=1&ave_thresh=50&_ignoreionsscorebelow=-1&report=0&_sigthreshold=0.05&_msresflags=1033&_msresflags2=256&percolate=-1&percolate_rt=0&_minpeplen=7&sessionID=Vaibhav_S_2095245552053707) | AGABI2DRAFT_192925 | 50,087 | 877 | 12(12) | 11(11) | 1.11 |
|  | [gi\|597968107](http://bio-mascot1.chem.umu.se/mascot/cgi/protein_view.pl?file=..%2Fdata%2F20190509%2FF116022.dat&hit=gi%7C597968107&db_idx=1&px=1&ave_thresh=50&_ignoreionsscorebelow=-1&report=0&_sigthreshold=0.05&_msresflags=1033&_msresflags2=256&percolate=-1&percolate_rt=0&_minpeplen=7&sessionID=Vaibhav_S_2095245552053707) | AGABI1DRAFT_11813 | 135,151 | 560 | 8(8) | 8(8) | 0.22 |
|  | [gi\|568434474](http://bio-mascot1.chem.umu.se/mascot/cgi/protein_view.pl?file=..%2Fdata%2F20190509%2FF116022.dat&hit=gi%7C568434474&db_idx=1&px=1&ave_thresh=50&_ignoreionsscorebelow=-1&report=0&_sigthreshold=0.05&_msresflags=1033&_msresflags2=256&percolate=-1&percolate_rt=0&_minpeplen=7&sessionID=Vaibhav_S_2095245552053707) | AGABI2DRAFT_189297 | 47,546 | 446 | 6(6) | 5(5) | 0.43 |
|  | [gi\|568442050](http://bio-mascot1.chem.umu.se/mascot/cgi/protein_view.pl?file=..%2Fdata%2F20190509%2FF116022.dat&hit=gi%7C568442050&db_idx=1&px=1&ave_thresh=50&_ignoreionsscorebelow=-1&report=0&_sigthreshold=0.05&_msresflags=1033&_msresflags2=256&percolate=-1&percolate_rt=0&_minpeplen=7&sessionID=Vaibhav_S_2095245552053707) | AGABI2DRAFT_195585 | 87,082 | 431 | 5(5) | 5(5) | 0.22 |
|  | [gi\|568458572](http://bio-mascot1.chem.umu.se/mascot/cgi/protein_view.pl?file=..%2Fdata%2F20190509%2FF116022.dat&hit=gi%7C568458572&db_idx=1&px=1&ave_thresh=50&_ignoreionsscorebelow=-1&report=0&_sigthreshold=0.05&_msresflags=1033&_msresflags2=256&percolate=-1&percolate_rt=0&_minpeplen=7&sessionID=Vaibhav_S_2095245552053707) | AGABI2DRAFT_193317 | 59,788 | 411 | 6(6) | 5(5) | 0.33 |
|  | [gi\|568460577](http://bio-mascot1.chem.umu.se/mascot/cgi/protein_view.pl?file=..%2Fdata%2F20190509%2FF116022.dat&hit=gi%7C568460577&db_idx=1&px=1&ave_thresh=50&_ignoreionsscorebelow=-1&report=0&_sigthreshold=0.05&_msresflags=1033&_msresflags2=256&percolate=-1&percolate_rt=0&_minpeplen=7&sessionID=Vaibhav_S_2095245552053707) | AGABI2DRAFT_193748 | 54,126 | 369 | 4(4) | 4(4) | 0.29 |
|  | [gi\|597966151](http://bio-mascot1.chem.umu.se/mascot/cgi/protein_view.pl?file=..%2Fdata%2F20190509%2FF116022.dat&hit=gi%7C597966151&db_idx=1&px=1&ave_thresh=50&_ignoreionsscorebelow=-1&report=0&_sigthreshold=0.05&_msresflags=1033&_msresflags2=256&percolate=-1&percolate_rt=0&_minpeplen=7&sessionID=Vaibhav_S_2095245552053707) | AGABI1DRAFT_110949 | 58,690 | 332 | 5(5) | 5(5) | 0.34 |
|  | [gi\|568443552](http://bio-mascot1.chem.umu.se/mascot/cgi/protein_view.pl?file=..%2Fdata%2F20190509%2FF116022.dat&hit=gi%7C568443552&db_idx=1&px=1&ave_thresh=50&_ignoreionsscorebelow=-1&report=0&_sigthreshold=0.05&_msresflags=1033&_msresflags2=256&percolate=-1&percolate_rt=0&_minpeplen=7&sessionID=Vaibhav_S_2095245552053707) | AGABI2DRAFT_195919 | 54,800 | 313 | 5(5) | 5(5) | 0.36 |
|  | [gi\|294346665](http://bio-mascot1.chem.umu.se/mascot/cgi/protein_view.pl?file=..%2Fdata%2F20190509%2FF116022.dat&hit=gi%7C294346665&db_idx=1&px=1&ave_thresh=50&_ignoreionsscorebelow=-1&report=0&_sigthreshold=0.05&_msresflags=1033&_msresflags2=256&percolate=-1&percolate_rt=0&_minpeplen=7&sessionID=Vaibhav_S_2095245552053707) | Tyrosinase Chain A | 66,494 | 303 | 4(4) | 4(4) | 0.23 |
|  | [gi\|568457784](http://bio-mascot1.chem.umu.se/mascot/cgi/protein_view.pl?file=..%2Fdata%2F20190509%2FF116022.dat&hit=gi%7C568457784&db_idx=1&px=1&ave_thresh=50&_ignoreionsscorebelow=-1&report=0&_sigthreshold=0.05&_msresflags=1033&_msresflags2=256&percolate=-1&percolate_rt=0&_minpeplen=7&sessionID=Vaibhav_S_2095245552053707) | AGABI2DRAFT_185824 | 82,636 | 219 | 3(3) | 3(3) | 0.13 |
|  | [gi\|568433906](http://bio-mascot1.chem.umu.se/mascot/cgi/protein_view.pl?file=..%2Fdata%2F20190509%2FF116022.dat&hit=gi%7C568433906&db_idx=1&px=1&ave_thresh=50&_ignoreionsscorebelow=-1&report=0&_sigthreshold=0.05&_msresflags=1033&_msresflags2=256&percolate=-1&percolate_rt=0&_minpeplen=7&sessionID=Vaibhav_S_2095245552053707) | AGABI2DRAFT_62224 | 44,382 | 189 | 2(2) | 2(2) | 0.16 |
|  | [gi\|568451418](http://bio-mascot1.chem.umu.se/mascot/cgi/protein_view.pl?file=..%2Fdata%2F20190509%2FF116022.dat&hit=gi%7C568451418&db_idx=1&px=1&ave_thresh=50&_ignoreionsscorebelow=-1&report=0&_sigthreshold=0.05&_msresflags=1033&_msresflags2=256&percolate=-1&percolate_rt=0&_minpeplen=7&sessionID=Vaibhav_S_2095245552053707) | AGABI2DRAFT_191507 | 68,424 | 68424 | 2(2) | 2(2) | 0.10 |
|  | [gi\|597986243](http://bio-mascot1.chem.umu.se/mascot/cgi/protein_view.pl?file=..%2Fdata%2F20190509%2FF116022.dat&hit=gi%7C597986243&db_idx=1&px=1&ave_thresh=50&_ignoreionsscorebelow=-1&report=0&_sigthreshold=0.05&_msresflags=1033&_msresflags2=256&percolate=-1&percolate_rt=0&_minpeplen=7&sessionID=Vaibhav_S_2095245552053707) | AGABI1DRAFT_114685 | 48,352 | 151 | 2(2) | 2(2) | 0.15 |
|  | [gi\|568444913](http://bio-mascot1.chem.umu.se/mascot/cgi/protein_view.pl?file=..%2Fdata%2F20190509%2FF116022.dat&hit=gi%7C568444913&db_idx=1&px=1&ave_thresh=50&_ignoreionsscorebelow=-1&report=0&_sigthreshold=0.05&_msresflags=1033&_msresflags2=256&percolate=-1&percolate_rt=0&_minpeplen=7&sessionID=Vaibhav_S_2095245552053707) | AGABI2DRAFT_196132 | 54,893 | 112 | 2(2) | 2(2) | 0.13 |
